# Detection of Modern Spinal Implants by Handheld Metal Detectors: A Prospective Observational Study

**DOI:** 10.7759/cureus.97443

**Published:** 2025-11-21

**Authors:** Bharat R Dave, Amritesh Singh, Ravi Ranjan Rai, Ajay Krishnan, Shivanand C Mayi, Mirant B Dave, Mikeson Panthackel, Arjit Vashishtha, Mahesh Sagar, Yogen Adodariya, Sandesh Subhas Agarawal

**Affiliations:** 1 Spine Surgery, Stavya Spine Hospital and Research Institute, Ahmedabad, IND; 2 Spine Surgery, Bhavnagar Institute of Medical Science (BIMS), Bhavnagar, IND

**Keywords:** airport security, handheld detectors, metal detection, spinal fusion, spinal instrumentation

## Abstract

Introduction

The proliferation of spinal fusion procedures over the past two decades has introduced various metallic implants, stainless steel, cobalt-chromium, and titanium alloys, that exhibit differential responses to security detection systems. Limited evidence exists regarding the detectability of spinal implants during airport security screening compared to joint replacements. Post-9/11 aviation security enhancements have intensified screening protocols, creating patient anxiety regarding potential alarm activation. This investigation sought to quantify spinal implant detection rates using handheld metal detection technology to establish evidence-based patient guidance and certification protocols.

Materials and methods

A prospective cohort study was implemented at Stavya Spine Institute and Research Center, Ahmedabad, encompassing 500 consecutive adult patients receiving instrumented spinal procedures. Study exclusions comprised previous spinal operations, ambulatory limitations, neurological compromise, non-instrumented surgeries, and concurrent metallic implants. Standardized screening occurred on postoperative day two and discharge using the GARRETT SuperWand® (Garrett Electronics Inc., Garland, TX) handheld detectors calibrated to maximum sensitivity. Participants donned institutional garments and removed all personal metallic items prior to assessment, replicating airport security protocols. Data acquisition included demographic characteristics, anthropometric measurements, operative details, hardware specifications, and detection outcomes. Spearman correlation analysis evaluated BMI-detection relationships.

Results

The study population included 271 females (54.2%) and 229 males (45.8%), with a mean age of 54.6 ± 14.4 years and an average BMI of 26.9 ± 5.0 kg/m². Anatomical distribution encompassed lumbar procedures 336 (67.2%), cervical operations 84 (16.8%), thoracic surgeries 41 (8.2%), and multilevel fusions 39 (7.8%). Detection occurred in 24 patients (4.8%) out of 500 total participants. Regional analysis demonstrated marked variability: thoracic procedures yielded 12 detections (29.3%) out of 41 cases, multilevel constructs six detections (15.4%) out of 39 cases, posterior cervical approaches four detections (15.4%) out of 26 cases, and lumbar surgeries two detections (0.6%) out of 336 cases. Anterior cervical procedures produced zero detections (0%) out of 58 cases. Among 2534 total screws, 223 posterior elements triggered detection (8.8% rate), while anterior hardware remained undetected (0%). BMI demonstrated a weak but significant correlation with detection probability (r = 0.136, p = 0.003).

Conclusion

Handheld metal detection identifies spinal instrumentation in 4.8% of cases, exhibiting pronounced anatomical heterogeneity in detection patterns. Posterior thoracic hardware presents maximum detection risk, while anterior cervical and routine lumbar constructs demonstrate minimal alarm probability. These findings endorse individualized patient counseling and selective documentation practices: patients with high-detection-risk implants should receive travel certificates to expedite security processing, whereas those with anterior cervical or standard lumbar hardware can be assured of negligible detection likelihood. This data-driven approach optimizes patient preparation for air travel while minimizing unnecessary administrative burden.

## Introduction

Over the past twenty years, spinal fusion for adolescent idiopathic scoliosis has grown increasingly common, and surgeons now choose from stainless steel, cobalt-chromium, or titanium alloys, each of which interacts differently with magnetic fields [1,2]. Whether an orthopedic device sets off a detector depends on its metal composition, overall size and mass, complexity of the construct, and where it lies on the body. Research on hip and knee replacements has shown detection rates anywhere from about one-third up to nearly 90%, depending on prosthesis type and security-system settings [3,4]. Yet these studies offer little insight into how spinal instrumentation behaves in airport checkpoints.

Airport security screening has become far more stringent since the September 11, 2001, attacks, with standard walkthrough metal detectors now tuned to much higher sensitivities and advanced imaging systems, such as millimeter-wave body scanners, deployed in many airports [5,6]. This has resulted in a greater incidence of false alarms for patients with metal implants from prior orthopedic procedures, most notably total joint ar­throplasty [7]. While these upgrades bolster overall safety, they also prompt worry among individuals with spinal hardware, who fear that their implants might activate alarms and lead to extra screening or travel disruptions. The increased rate of false alarms for orthopedic patients leads to anxiety and uncertainty of unexpected travel de­lays and more extensive searches [8]. Total joint prostheses will routinely set off the detector, whereas nails, plates, screws, and wires are rarely detected. Cobalt-chromium and titanium implants are more likely to be detected than stainless-steel implants [9].

Few reports address spinal implants specifically. In the United Kingdom, a study of forty patients fitted with titanium-only spinal constructs found no airport alarms triggered by their hardware [10]. Conversely, a pediatric series in the United States observed that nearly a quarter of children with cobalt-chromium rods underwent additional screening, while none with stainless-steel rods did [1]. This apparent contradiction highlights the need for a clear, evidence-based understanding of how implant metallurgy, construct design, and modern screening technologies combine to influence detection.

Arming families with this information allows surgeons to set accurate expectations. Patients can better prepare for travel -- knowing whether to carry detailed implant documentation, anticipate secondary screening, or choose certain alloy systems -- thus minimizing anxiety and ensuring smoother journeys.

As most of the airports use handheld metal detectors, the purpose of this study setup was to evaluate whether spine implants are routinely detected by handheld metal detectors, which can help doctors to advise and educate their patients accurately and in deciding whether they should routinely supply them with documentary evidence of their implant.

## Materials and methods

An observational study was conducted at the Stavya Spine Institute and Research Center, Ahmedabad, Gujarat. Institutional Ethics Committee approval was obtained (CTRI No. CTRI/2024/08/072947). A total of 500 patients who underwent surgery at the institute were enrolled in the study. The patients were admitted and operated on for their respective spinal pathologies according to institutional protocols. They were informed beforehand about the study, and written informed consent was obtained. The aim of the study was to determine whether spinal implants are routinely detected by hand-held metal detectors. 

The objectives of this prospective observational study were as follows: (1) To quantify the overall detection rate of spinal implants by handheld metal detectors; (2) To evaluate the region-specific detection rate of spine implants; and (3) To correlate the BMI of the patients with the overall detection rate of spine implants.

Achieving the above objectives will help doctors to advise and educate their patients accurately regarding the detection sensitivity of spinal implants and in deciding whether they should routinely supply them with documentary evidence of their implant.

Inclusion or exclusion from the study did not affect the patients' treatment. Patients included in the study were aged > 18 years and undergoing spinal surgery with instrumentation. Patients with previous spine surgery, non-ambulatory patients, patients with neurological deficit, patients undergoing non-instrumented surgeries, and patients who are not willing to give consent to participate in the study, and patients with pacemakers or other implanted metallic hardware that can interfere with the detection of spinal implants were excluded.

**Figure 1:**
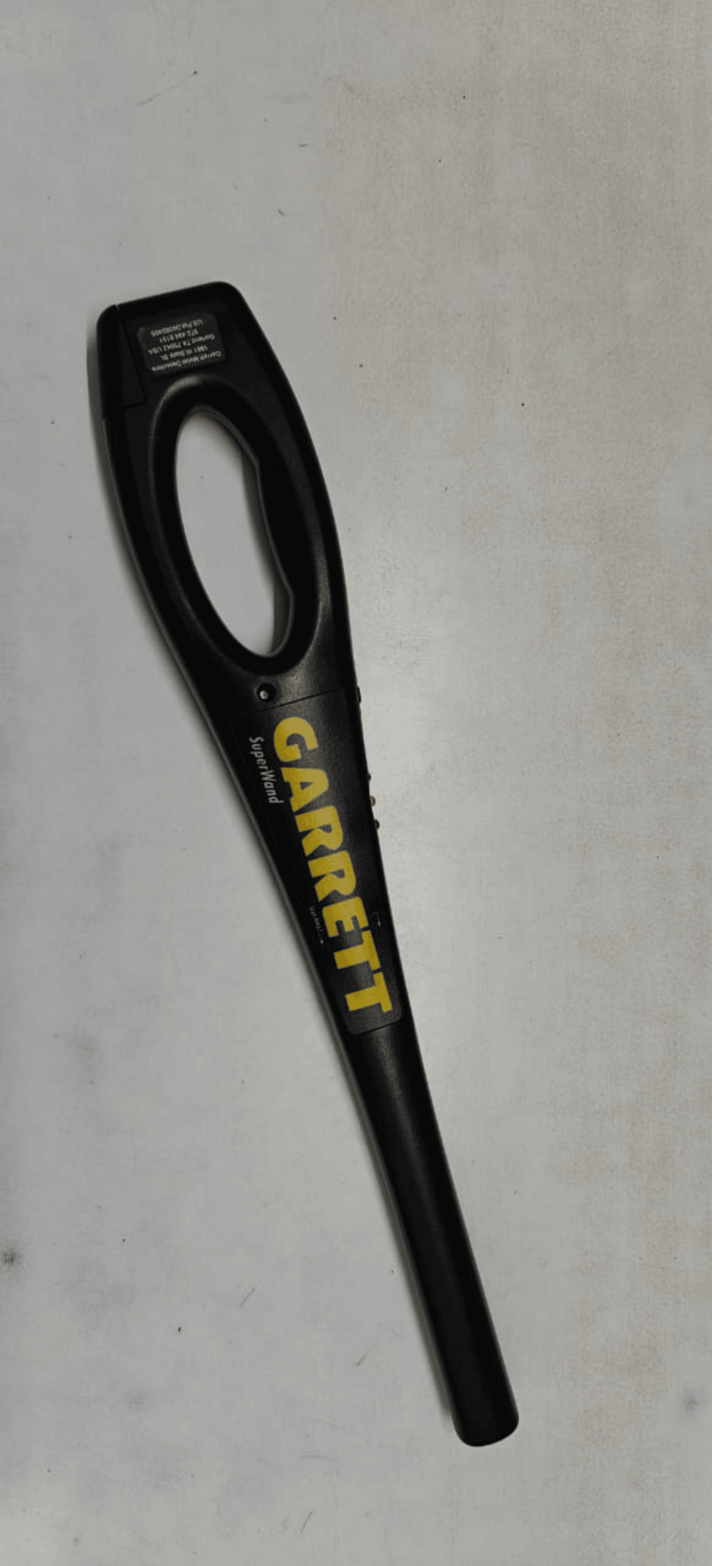
Image showing GARRETT SuperWand® handheld metal detector.

The patients were admitted through OPD for their respective pathologies, and detailed demographic and clinical information was recorded. Surgeries were performed by open or minimally invasive approach with or without navigation, as indicated for every patient. The titanium implants, screws, and cages were, in the majority, manufactured by titanium-aluminium-vanadium alloy. The connecting rods used were titanium or cobalt-chromium alloy. The implants for disc replacements were made from cobalt-chrome-molybdenum alloy. GARRETT SuperWand® (Garrett Electronics Inc., Garland, TX) was the device used for the study, as depicted in Figure 1.

**Figure 2:**
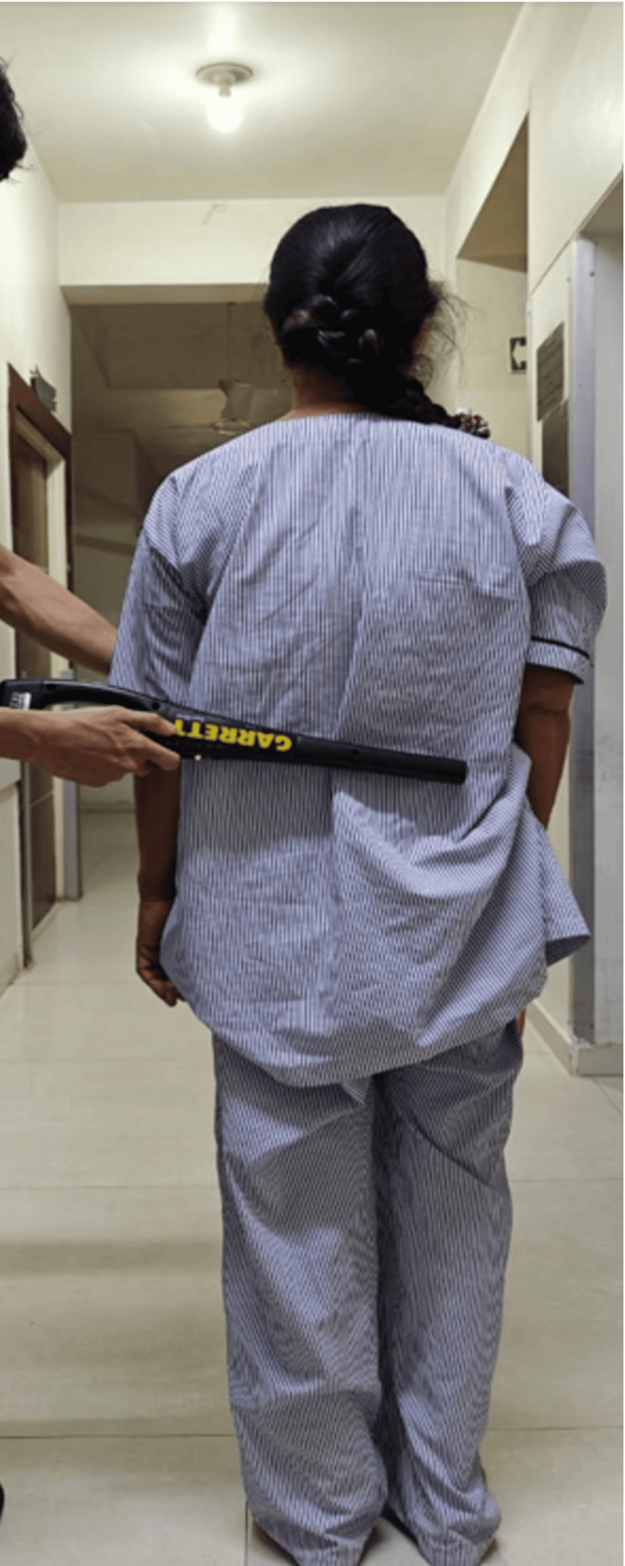
Image demonstrating the process of frisking in patients.

**Figure 3:**
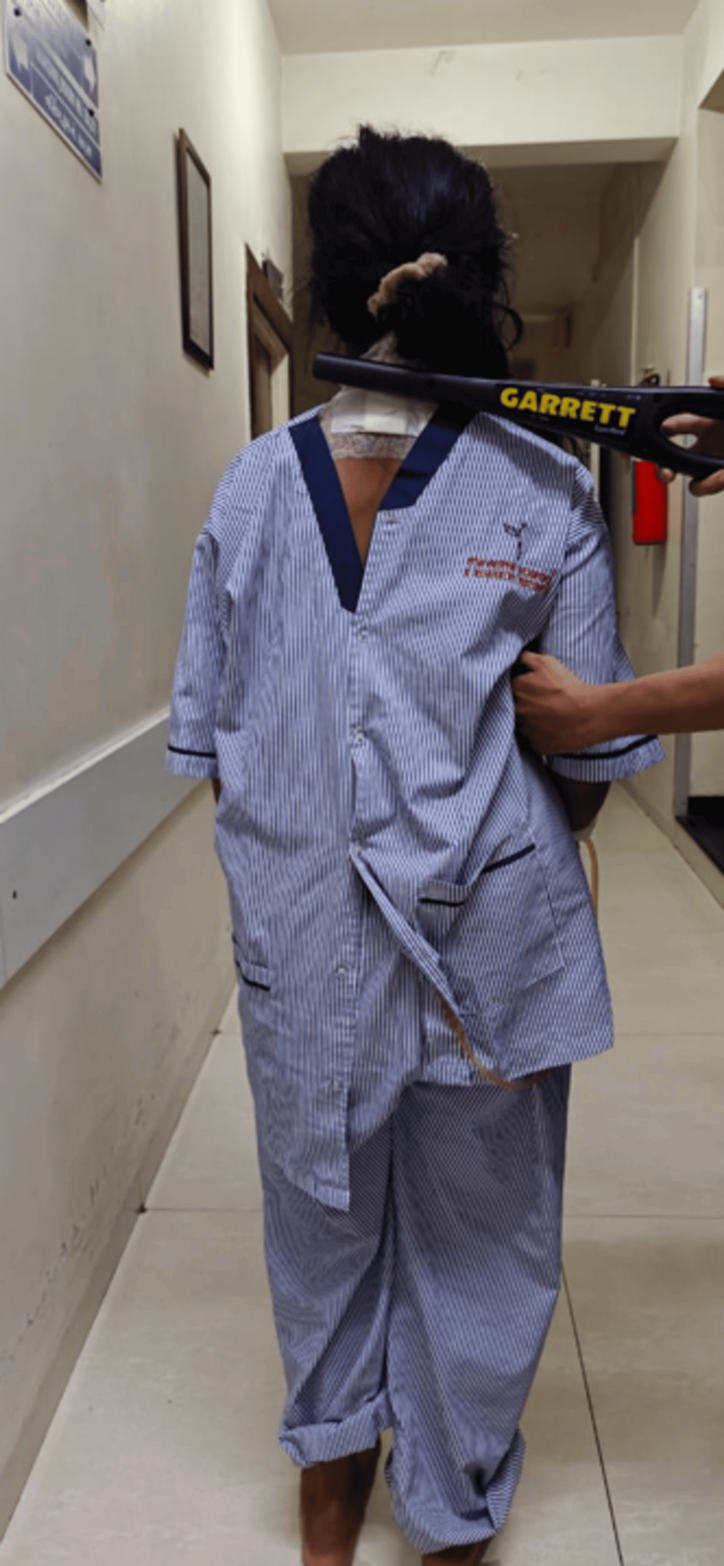
Image showing detection of implants in a cervical (posterior) case.

The device is self-calibrating, using digital microprocessor technology that automatically calibrates its sensitivity for optimal performance and eliminates the need for manual sensitivity adjustments. All the triggered alarms were noted. Frisking and sweeping of the operative site was done with a handheld metal detector on the second post-op day, when the patient was ambulated, as well as on the day of discharge, with the patient in an upright position. During this time, all patients were asked to remove all jewelery items, watches, belts, etc., as per the airport security protocols, and the patients were advised to be in light clothing provided by the hospitals. All patients were screened for metal detection by the same individual with appropriate training in the device usage. Shoes were removed, and females were asked to wear brassieres with no metal supports, as depicted in Figures 2, 3.

The data recorded was thus entered into a Microsoft Excel (Microsoft Corporation, Redmond, WA) spreadsheet for further analysis. Data recorded includes: (1) demographic data; (2) BMI; (3) diagnosis and procedure undergone; (4) whether or not the implants were detected on frisking.

The data obtained were entered into an Excel spreadsheet and subjected to statistical analysis using SPSS software (version 21.0) (IBM Corp., Armonk, NY). The demographic data would be expressed as mean and standard deviation. Based on the data, we would analyze the overall detection rate as well as the region-specific (cervical-anterior, cervical-posterior, dorsal, lumbar, >1 region) detection rates of spine implants in patients, which will be expressed as % age. The correlation between the BMI and the overall detection rate would be performed by the Spearman correlation test. The p-value of <0.05 will be considered as significant.

## Results

**Figure 4:**
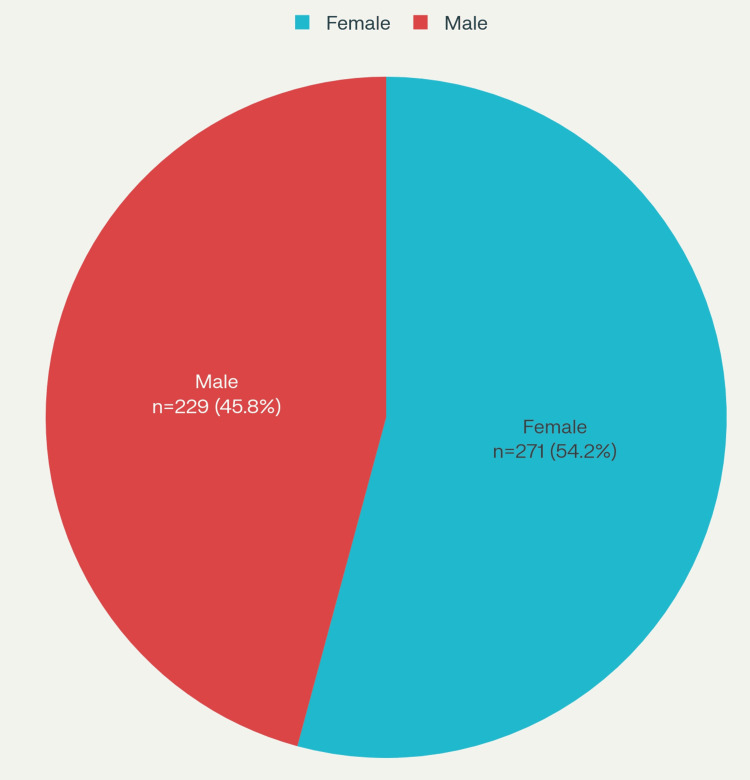
Gender distribution of cases.

**Figure 5:**
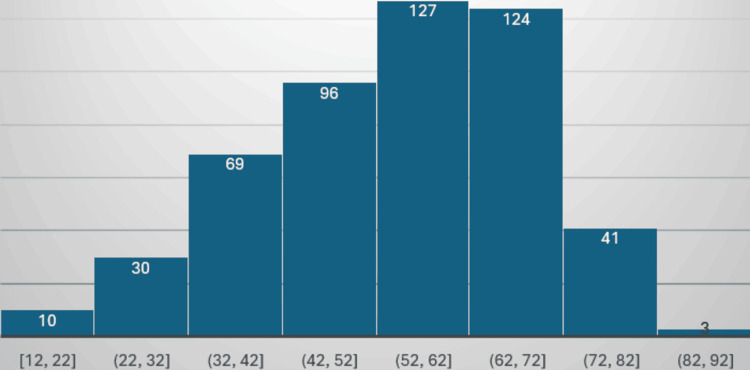
Age distribution of cases.

**Figure 6:**
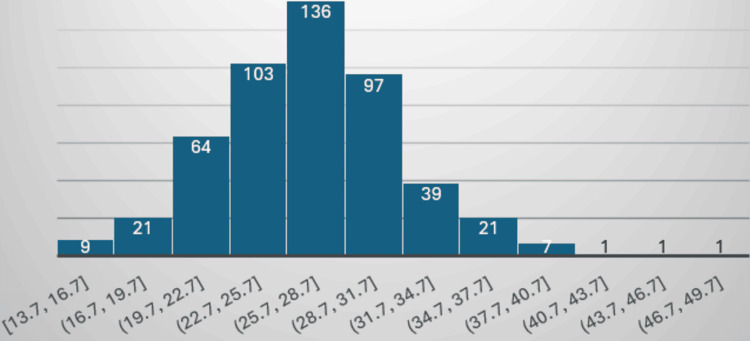
BMI distribution of cases.

**Figure 7:**
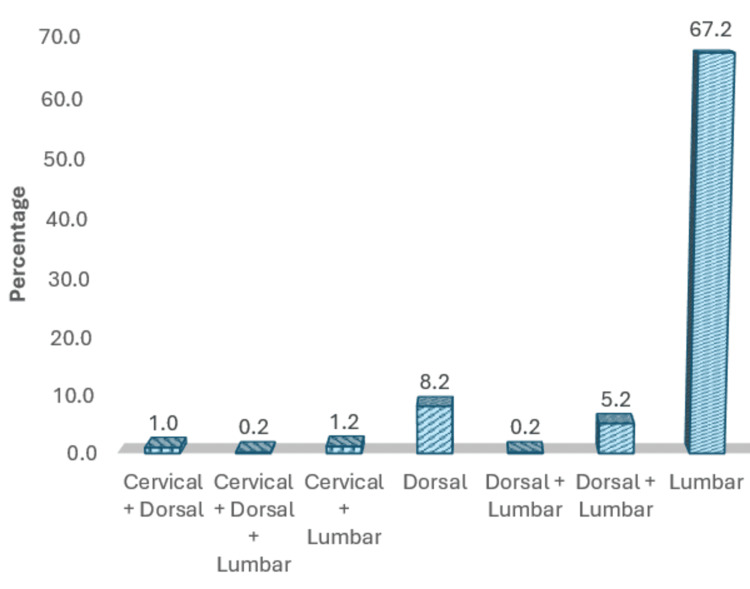
Region-specific distribution of operated cases.

The current study comprises 229 males (45.8%) and 271 females (54.2%), accounting for a total of 500 patients, as shown in Figure 4. The mean age of patients is 54.55 ± 14.4 years (12-87 years) as depicted in Figure 5. The mean BMI is 26.9 ± 5.03 kg/m² (13.7-47.9 kg/m²) as depicted in Figure 6. The age and BMI characteristics are tabulated in Table 1. Out of 500 patients, 336 (67.2%) are operated for lumbar spine, 84 (16.8%) for cervical spine, 41 (8.2%) for dorsal spine, 27 (5.4%) for dorsal + lumbar, 6 (1.2%) for cervical + lumbar, 5 (1.0%) for cervical + dorsal, and 1 (0.2%) for cervical + dorsal + lumbar spine as depicted in Figure 7. Of the patients who underwent cervical spine surgery, 58 cases (69.0%) were operated via the anterior approach, while 26 cases (31.0%) were operated via the posterior approach.

**Table 1:** Age and BMI characteristics of patients.

Parameter	Mean ± SD	Range
Age	54.55 ± 14.4 years	12-87 years
BMI	26.9 ± 5.03 kg/m^2^	13.7-47.9 kg/m^2^

**Figure 8:**
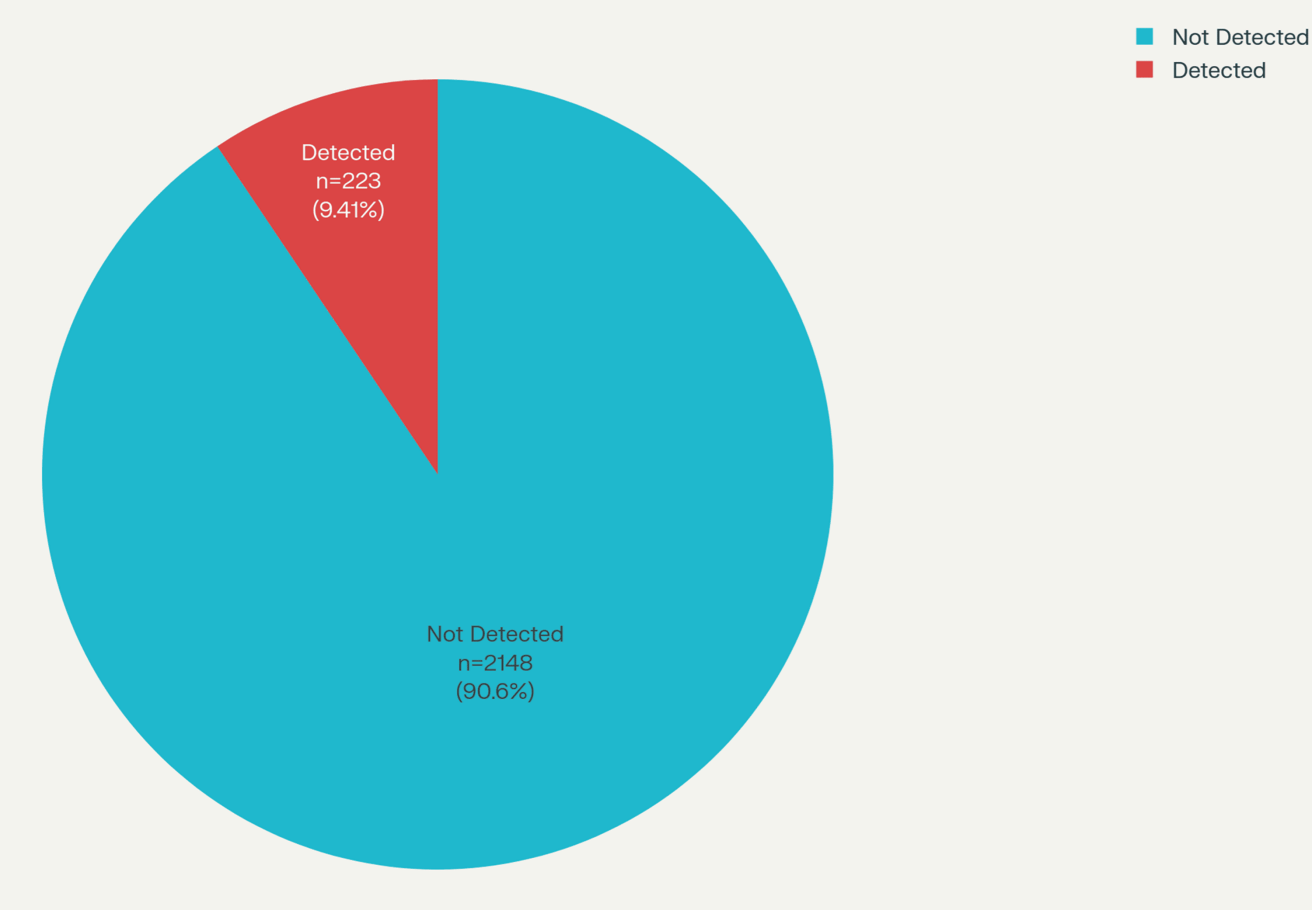
Graph showing the percentage of posterior screws detected.

On using a handheld metal detector on the patients, 24 out of 500 patients (4.8%) were found to be positive, thereby giving us an overall detection rate of 4.80%. On the subset analysis, detection rate was 12 out of 41 patients (29.3%) for dorsal spine operated patients, six out of 26 patients (23.1%) for dorsal + lumbar spine operated patients, two out of 336 patients (0.59%) for lumbar spine operated patients, and four out of 84 patients (4.8%) for cervical (overall) operated patients. The above data are tabulated in Table 2. No implants were detected in 58 patients (0%) operated for cervical spine who were approached anteriorly. A total of 2534 screws were utilized in the patients, out of which 2371 screws (93.6%) were utilized in the cases approached posteriorly, while 163 screws (6.4%) were used in anterior approaches, as tabulated in Table 3. The handheld metal detector was able to detect 223 out of 2371 screws (9.4%) used during posterior approach, as depicted in Figure 8, while 0 out of 163 screws (0%) were detected in anterior approaches. Table 4 depicts the individual values of BMI and the number of screws utilized in 24 positive detected patients (100%).

**Figure 9:**
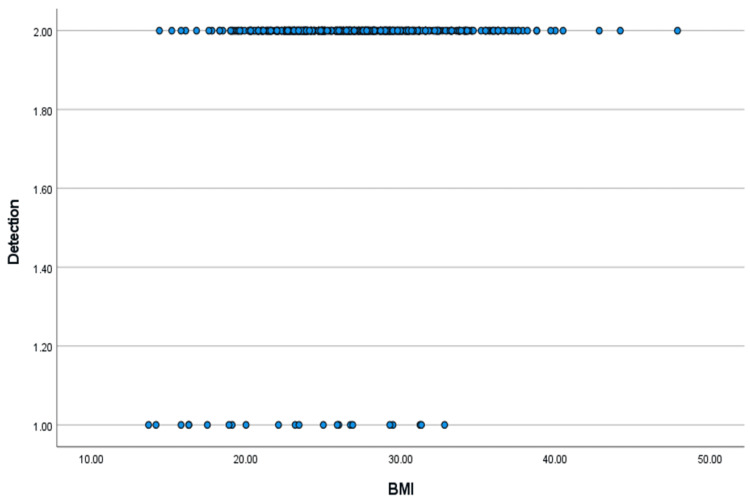
Graph showing the Spearman's correlation between BMI and the overall detection rate.

Using Spearman's correlation coefficient, the correlation between the detection rate and the BMI was found to be 0.136 with a p-value of 0.003. The data are depicted in Table 5 and Figure 9.

**Table 2:** Table demonstrating the subset and overall detection rate of spinal implants.

Region operated	No. of patients	Positive detection	Subset detection rate (%)	Overall detection rate (%)
Cervical (anterior)	58	0	0	0
Cervical (posterior)	26	4	15.38	0.8
Dorsal	41	12	29.3	2.4
Lumbar	336	2	0.59	0.4
>1 region operated	39	6	15.38	1.2
Total patients operated	500	24	Overall detection rate = 4.80%	

**Table 3:** Table showing the number of screws and their percentage detection by handheld metal detectors.

Parameter	Value
Total no. of screws used (posterior + anterior)	2534
Total no. of screws used (posterior only)	2371
Total no. of screws used anteriorly	163
Posterior screws detected positive using handheld metal detector	223
Anterior screws detected using handheld metal detector	0
% Detection (posterior)	9%
% Detection (anterior)	0

**Table 4:** Table showing the characteristics of positive detected cases.

Serial no. of patients with positive detection rate	Region operated	Approach	BMI	No. of screws
1	D2–L3	Posterior	16.3	16
2	D2–L4	Posterior	18.9	18
3	C1–C2	Posterior	16.3	4
4	C1–C2	Posterior	19.1	4
5	D2–L1	Posterior	13.7	12
6	D5–L1	Posterior	22.8	11
7	D5–L1	Posterior	22.1	12
8	D11–L1	Posterior	15.8	5
9	D3–D12	Posterior	29.5	20
10	D6–D10	Posterior	17.5	8
11	D8–D11	Posterior	20	8
12	D11–L4	Posterior	29.3	8
13	D2–D12	Posterior	23.18	12
14	D5–D9	Posterior	27.1	7
15	D10–S1	Posterior	23.43	11
16	Occiput–C5	Posterior	26	6
17	L1–S1	Posterior	31.34	8
18	L1–L5	Posterior	31.25	10
19	D12–L2	Posterior	26.9	6
20	C4–C5	Posterior	26.75	4
21	D10–D12	Posterior	25	6
22	D9–D12	Posterior	32.84	7
23	D5–D8	Posterior	25.9	8
24	D4–L3	Posterior	14.18	12

**Table 5:** Spearman correlation test between BMI and the overall detection rate of implants.

Spearman's correlation			
BMI and overall detection rate	Correlation coefficient	0.136
	P-value	0.003
	N	474

## Discussion

The detection of spinal implants by airport security systems represents an important clinical concern that has gained prominence since the implementation of enhanced security measures following the September 11, 2001, incident. The evolution from traditional walkthrough metal detectors to advanced imaging technologies, including millimeter-wave body scanners, has fundamentally altered the detection landscape for patients with orthopedic implants. Current scientific evidence reveals significant variability in spinal implant detection rates, ranging from 4.8% to 86% depending on the screening technology employed and study methodology.

The present study was conducted at Stavya Spine Institute, Ahmedabad. With a robust sample size of 500 patients, comprising a majority of females and a mean BMI of 26.9 ± 5.03 kg/m^2^ (13.7-47.9 kg/m^2^), the study used a handheld metal detector set to high sensitivity and documented an overall detection rate of 4.8% among 500 patients. This finding contrasts markedly with recent reports showing substantially higher rates when full-body scanners are employed. Arhewoh et al. recently demonstrated that 40% of patients with spinal implants experienced detector alerts at airports, with a notable difference between screening technologies: full-body scanners detected implants in 86% of cases compared to 54% for traditional metal detectors [8]. This represents a dramatic increase from historical studies, which reported detection rates as low as 5.6% in pediatric populations [2]. The discrepancy between these findings underscores the critical importance of distinguishing between different screening technologies when interpreting detection data and counseling patients.

The variability extends to anatomical location, with the current study showing dorsal spine implants achieving the highest detection rate at 29.3% (n = 12), followed by multilevel constructs at 15.38% (n = 6), while anterior cervical approaches demonstrated no detections. This pattern aligns with findings from Chinwalla and Grevitt, who observed that enhanced metal detectors failed to detect any modern spinal implants, while handheld detectors successfully identified all posterior instrumentation [6]. The complete absence of detections in anterior cervical procedures (0 out of 58 cases) aligns with previous reports demonstrating the distance-dependent nature of metal detector sensitivity. The deeper anatomical location of anterior cervical instrumentation, combined with tissue masking effects, appears to render these constructs undetectable by handheld screening devices at standard operating distances.

The influence of implant material composition on detection rates remains one of the most investigated yet controversial aspects of airport security screening. Woon et al. provided compelling evidence that cobalt-chromium constructs trigger additional screening in 24% of pediatric patients, while stainless-steel constructs showed zero detection rates [1]. These finding challenges traditional assumptions about ferromagnetic materials being more readily detectable. Kunasuntiwarakul and Poopitaya reported that 93% of spine implants were detected using handheld metal detectors, with cobalt-chromium implants being twice as likely to be detected compared to stainless steel [9]. Their comprehensive analysis of 386 implants demonstrated that material composition serves as an independent predictor of detection, with cobalt-chromium and titanium implants showing higher detection rates than stainless-steel constructs. In our study, we have tested a total of 2534 screws, out of which only 223 were detected by handheld detectors.

However, these findings contrast with earlier reports from Fabricant et al., who observed that only stainless-steel instrumentation triggered metal detectors in pediatric patients, with all titanium constructs remaining undetected [2]. The temporal aspect of this study is particularly noteworthy, as all positive detections occurred in patients with stainless-steel instrumentation implanted prior to 2008, suggesting potential evolution in manufacturing processes or alloy composition. The current study's finding of a weak but statistically significant correlation between BMI and detection rate (r = 0.136, p = 0.003) contradicts several earlier investigations that found no BMI effect. This discrepancy may reflect differences in study populations, detector types, or statistical methodology.

The introduction of millimetre-wave body scanners has fundamentally altered the detection paradigm for spinal implants. Unlike traditional pulse induction metal detectors, which rely on electromagnetic field disturbances, advanced imaging systems operate on different physical principles and demonstrate markedly different sensitivity patterns. Obremskey et al. established benchmark data using TSA-certified equipment, demonstrating that prostheses, plates longer than 10 holes, and titanium nails were the strongest predictors of detection by walkthrough metal detectors [5]. Their systematic analysis revealed that body mass index did not significantly affect detection rates, contrary to earlier hypotheses about tissue masking effects.

The evolution of screening technology has created a complex detection landscape where handheld metal detectors demonstrate superior sensitivity compared to walkthrough systems. Chinwalla and Grevitt found that handheld devices detected all posterior spinal implants at distances of 5 cm, while walkthrough detectors failed to identify any constructs despite titanium masses up to 215 grams [6]. This finding has important practical implications, as patients who do not trigger walkthrough detectors may still be identified during secondary screening with handheld devices if subjected to additional scrutiny for other reasons.

Beyond the technical aspects of detection, the psychological and practical implications for patients with spinal implants cannot be understated. The uncertainty surrounding potential detection creates significant anxiety for travelers, particularly those who have never experienced airport security screening with their implants. Fabricant et al. noted that the potential for family separation during additional screening procedures represents a particular concern for pediatric patients and their families [2]. The emotional impact extends beyond inconvenience, as patients report feeling stigmatized or embarrassed during secondary screening procedures. Studies consistently demonstrate that the majority of detected patients (72-96%) experience only minor inconvenience, requiring simple explanations or brief additional screening [4,10]. However, the unpredictability of detection creates a psychological burden that affects travel planning and decision-making for many patients.

Recent evidence suggests that patient education and preparation significantly improve the airport security experience. Arhewoh et al. found that patients who were informed about potential detection and prepared with appropriate documentation reported less stress and more positive interactions with security personnel [8]. This finding emphasizes the critical role of surgeons in providing accurate, evidence-based counseling during preoperative discussions. The current study's regional variation in detection rates provides important insights for surgical planning and patient counseling, with dorsal spine constructs demonstrating the highest detection rates (29.3%, n = 12), likely reflecting the superficial position of posterior instrumentation and the typically larger metal mass associated with pedicle screw systems.

The utility and necessity of medical documentation for patients with spinal implants remains a subject of ongoing debate within the orthopedic community. Traditional practice has often included the provision of implant identification cards or medical certificates detailing the presence and characteristics of spinal hardware [11-13]. However, recent evidence questions the practical value of such documentation. One plausible solution was presented by Fong et al. [14] describing the use of a biomet­ric medical card that contains the patient's medical history for user identity authentication. The use of such technology could give a reliable method of confirming orthopedic implantation during airport screening. Ali et al. conducted a comprehensive survey of both patients and airport security officers, finding that 84% of patients and 90% of security personnel believed implant identification cards would streamline security processes [10]. Their study demonstrated that documentation was perceived as beneficial by both parties, potentially reducing the need for invasive secondary screening procedures.

Conversely, Fabricant et al. reported that even when patients presented implant cards during detection episodes, they were still subjected to additional screening procedures, suggesting that documentation does not provide exemption from security protocols [2]. The Transportation Security Administration's policy explicitly states that medical documentation does not exempt travelers from additional screening if an alarm is triggered, though it may help explain the presence of implants and facilitate communication with security personnel. The current evidence suggests a nuanced approach to documentation, where provision of medical certificates may be most beneficial for frequent travelers, patients with large construct systems, or those with anxiety about potential detection. For the general patient population, given the relatively low overall detection rates demonstrated in multiple studies, routine provision of documentation may not be necessary.

Emerging evidence suggests that patient age may influence detection rates, though the mechanisms underlying this relationship remain unclear. Arhewoh et al. identified a significant correlation between patient age and positive screenings, with patients over 60 years demonstrating significantly higher detection rates than younger cohorts [8]. This finding raises the possibility that concurrent total joint arthroplasties in older patients may contribute to positive screening results, as the mean age of patients with joint replacements was 65.5 years in their study. The age-related detection pattern has important implications for patient counseling, as older patients may require more detailed discussion of potential screening issues and more comprehensive preparation for travel.

Multilevel constructs showed intermediate detection rates (15.38%, n = 6) in the current study, suggesting that construct complexity and total metal volume may influence detection probability independently of anatomical location. This finding has implications for surgical decision-making in patients with high travel requirements, where minimizing construct complexity might reduce detection likelihood. The rapid evolution of both implant technology and security screening systems necessitates ongoing research to maintain current evidence-based recommendations. The development of newer implant materials, including polyetheretherketone (PEEK) components and bioabsorbable materials, may fundamentally alter detection patterns compared to traditional metallic constructs.

Based on the current scientific evidence, several clinical recommendations emerge. Surgeons should provide individualized counseling based on implant characteristics rather than universal recommendations. Patients with posterior thoracic constructs or multilevel instrumentation should be advised of higher detection probability, while those with anterior cervical hardware can be reassured of minimal detection risk. The provision of medical documentation should be considered for frequent travelers, patients with anxiety about screening procedures, or those with large construct systems. While documentation does not exempt patients from additional screening, it may facilitate communication with security personnel and reduce procedural delays.

Patient education should emphasize that detection, when it occurs, typically results in minor inconvenience rather than significant travel disruption. Most detected patients require only brief additional screening with handheld devices, and reports of substantial delays or invasive procedures are rare in the literature, as mentioned in a study by Dines et al. [15]. The scientific literature on spinal implant detection by airport security systems reveals a complex landscape influenced by screening technology, implant characteristics, anatomical factors, and patient demographics. While detection rates vary substantially across studies, the overall evidence suggests that most patients with spinal implants can travel without significant security-related complications. The key to optimal patient care lies in providing accurate, evidence-based counseling that considers individual risk factors and helps patients make informed decisions about travel preparation and documentation needs.

This study's primary strength lies in its large, unselected cohort of 500 consecutive patients undergoing instrumented spinal surgery, reflecting real-world clinical practice and a broad spectrum of pathologies, implant types, anatomical levels, and surgical approaches. By using a standardized, high-sensitivity handheld metal detector across every patient in a single institution, the investigation minimizes variability in screening technique and detector settings. Detailed recording of demographic data, body mass index, implant metallurgy, construct complexity, and anatomical location enables rigorous sub-analysis of factors influencing detectability.

However, limitations must be acknowledged. First, only handheld detectors were employed; patients in actual airports typically encounter walkthrough arch detectors first, and detection patterns may differ between device types. Second, this single-center study may not capture regional variations in screening protocols or detector calibration. Thirdly, the variations in the demographic profile of the sample set also plays a role in variability of detection sensitivity. Finally, reliance on a single postoperative evaluation does not account for potential changes in implant position or body habitus over time.

## Conclusions

This prospective cohort study demonstrates notable variability in the sensitivity of handheld metal detectors for spinal implants, influenced by anatomical location, implant positioning, and implant characteristics. Detection rates were higher for posterior and multilevel constructs, likely due to superficial positioning and greater metal content, whereas anterior cervical and uncomplicated lumbar implants showed minimal detectability. These patterns support tailored clinical protocols for patient preparation based on implant location.

Furthermore, patient-specific factors, such as body mass index, modestly affected detection likelihood, reinforcing the need for individualized risk assessment. Collectively, the findings advocate for risk-stratified documentation practices, patients with implants are less likely to trigger detectors, and may forgo routine certification, while those with posterior or complex constructs should obtain documentation to facilitate efficient security screening.
